# Internet Cognitive-Behavioral Therapy for Painful Chronic Pancreatitis: A Pilot Feasibility Randomized Controlled Trial

**DOI:** 10.14309/ctg.0000000000000373

**Published:** 2021-06-18

**Authors:** Tonya M. Palermo, Emily F. Law, Mark D. Topazian, Katherine Slack, Blake F. Dear, Yeon Joo Ko, Santhi Swaroop Vege, Evan Fogel, Guru Trikudanathan, Dana K. Andersen, Darwin L. Conwell, Dhiraj Yadav

**Affiliations:** 1 Department of Anesthesiology and Pain Medicine, University of Washington School of Medicine Seattle, Washington, USA;; 2 Center for Child Health, Behavior, and Development, Seattle Children's Research Institute, Seattle, Washington, USA;; 3 Division of Gastroenterology & Hepatology, Mayo Clinic, Rochester, Minnesota, USA;; 4 Department of Psychology, Macquarie University, Sydney, Australia;; 5 Division of Gastroenterology and Hepatology, Indiana University, Indianapolis, Indiana, USA;; 6 Division of Gastroenterology, Hepatology and Nutrition, University of Minnesota, Minneapolis, Minnesota, USA;; 7 Division of Digestive Diseases and Nutrition, National Institute of Diabetes and Digestive and Kidney Diseases, Bethesda, Maryland, USA;; 8 Division of Gastroenterology, Hepatology, and Nutrition, Ohio State University, Columbus, Ohio, USA;; 9 Division of Gastroenterology & Hepatology, University of Pittsburgh Medical Center, Pittsburgh, Pennsylvania, USA.

## Abstract

Severe abdominal pain is a cardinal symptom of chronic pancreatitis (CP) associated with a high economic and societal burden. In other chronic pain conditions, cognitive-behavioral therapy (CBT) has demonstrated efficacy in improving patient outcomes (e.g., pain-related disability and depression). However, CBT has not yet been evaluated in adult patients with painful CP. We aimed to (i) evaluate the feasibility and acceptability of an adapted Internet CBT program for CP and (ii) generate pilot data regarding the effects of treatment on patient pain outcomes.

**METHODS:**

Thirty adults (mean age = 49.8 years, SD = 12.5; 80% women) with suspected or definite CP were randomized to Internet CBT (Pancreatitis Pain Course) versus control. The Pancreatitis Pain Course has 5 CBT lessons (e.g., thought challenging, relaxation, and activity pacing) delivered over 8 weeks. Pain interference, pain intensity, and quality of life were assessed at pretreatment, posttreatment, and the 3-month follow-up. Qualitative interviews were conducted at posttreatment with a subset of participants.

**RESULTS:**

Eighty percent of participants rated the program as highly acceptable; 64.3% completed all 5 lessons. Qualitative data revealed positive perceptions of program features, relevancy, and skills. Patients randomized to Internet CBT demonstrated moderate to large effects in reducing pain intensity and pain interference from baseline to 3 months. The proportion of treatment responders (>30% improvement) was significantly greater in the Internet-CBT group than in the control group (50% vs 13%, Fisher exact *t* test *P* = 0.04).

**DISCUSSION:**

In this first trial of CBT pain self-management in CP, feasibility, acceptability, and preliminary efficacy for reducing pain and disability were demonstrated. Future definitive trials of CBT are needed.

## INTRODUCTION

Pancreatitis is a leading cause of hospitalizations for gastrointestinal disorders and a significant source of morbidity, mortality, and reduced quality of life (1–3). Severe abdominal pain is a cardinal symptom of pancreatitis, present in 85% of patients with chronic pancreatitis (CP) (4), and is associated with a high economic and societal burden. Pancreatitis costs the United States more than $2 billion/year in healthcare spending (3). As pain becomes more frequent and severe, it reduces health-related quality of life (HRQoL) across multiple domains of physical, psychological, and social functioning (5,6).

CP pain management is difficult. Patient response to non-narcotic medications and endoscopic and surgical interventions is often unpredictable and short-lasting. In clinical practice, painful CP is oftentimes managed with chronic opioids (7), which unfortunately have limited efficacy and substantial risk for adverse side effects. Although a challenge to manage clinically, pain management is a top priority of patients with CP.

Recent guidelines encourage the use of behavioral interventions as part of a multidisciplinary approach to managing painful CP (8). Pain self-management programs using cognitive-behavioral interventions (CBT) equip patients with coping skills to minimize the impact of painful conditions on activity participation and psychosocial well-being. Indeed, multiple systematic reviews and meta-analyses of CBT interventions for chronic pain demonstrate beneficial effects on pain, disability, HRQoL, and mood in many populations (9). Given the persistence of pain and its impacts, CBT could be of a significant benefit to patients with CP. There is some evidence that patients with CP will engage in mindfulness (10); however, to our knowledge, there are no published trials evaluating the feasibility, acceptability, or efficacy of pain self-management interventions for adults with painful CP.

Despite the efficacy of pain self-management programs, patient level (e.g., geographic restrictions) and system barriers (e.g., small number of trained clinicians available to deliver the therapies (11,12)) prevent many individuals from receiving treatment. To address these barriers, digital health interventions delivered via Internet or mobile applications have been developed and tested, expanding opportunities for intervening with patients remotely. There is now strong evidence for Internet-delivered chronic pain self-management interventions in both adult and pediatric populations (13) with patients showing improvements in pain and disability.

The *Pain Course* is an Internet-delivered pain self-management program demonstrating feasibility across numerous clinical settings, acceptability to individuals with chronic pain (e.g., arthritis, cancer, and musculoskeletal pain), and efficacy in improving relevant patient-reported outcomes (e.g., disability and depression) (14,15). Building from this research, we adapted the *Pain Course* to be relevant for use in adult patients with CP. Our primary aim was to evaluate the feasibility and acceptability of the adapted program in a pilot randomized controlled trial (RCT) with 2 conditions: Internet CBT versus a wait list control condition. We hypothesized that treatment feasibility would be demonstrated through favorable study recruitment/enrollment statistics, completion of lessons, and completion of telephone calls with study coaches. We also expected that participants would rate the intervention as highly acceptable on self-report measures and via qualitative interview. Our secondary aim was to generate pilot data regarding the effects of treatment on pain interference, pain intensity, and HRQoL from pretreatment to posttreatment and the 3-month follow-up.

## METHODS

### Participants and setting

The clinical trial was registered before initiating study enrollment (http://ClinicalTrials.gov; Identifier: NCT03322644). Participants were recruited from March 2018 to December 2019 from outpatient pancreas disease clinics at one of 5 participating Chronic Pancreatitis, Diabetes, and Pancreatic Cancer (CPDPC) sites: Indiana University, Mayo Clinic, University of Minnesota, University of Pittsburgh Medical Center, or Ohio State University, who are part of the NIH U01-sponsored Consortium to study CPDPC. Participants were also recruited from the community via a nationwide recruitment campaign delivered on the social media channels of the National Pancreas Foundation (NPF). The study was approved by the primary site's Institutional Review Board and the Institutional Review Board at each referring center. Participants gave informed consent before any research procedures.

### Recruitment

Providers at referring outpatient pancreas disease clinics gave potential participants a flyer about the study and asked if they would be willing to be contacted by study staff to undergo additional screening. Providers then sent potential participants' contact information and confirmation of their diagnosis to study staff via a secure study website or email.

To reach potential participants in the community, we partnered with the NPF to conduct a nationwide recruitment campaign using their established social media channels. The NPF advertised the study via postings on their website, e-Newsletter, Facebook, Twitter, Instagram, and LinkedIn accounts. Potential participants then contacted study staff via a toll-free phone number or email address provided in the study advertisement. For participants recruited from the community, their treating physician completed a medical history form to provide confirmation of their CP diagnosis before study enrollment.

Study staff screened all potential participants (referred from clinics and the community) by phone and then obtained verbal informed consent for study participation.

### Inclusion/exclusion criteria

Inclusion criteria were (i) age older than 18 years, (ii) meeting CPDPC criteria for diagnosis of either suspected (i.e., early stage) or definite CP as previously described (16), (iii) having personal Internet access on any device (e.g., phone and computer), and (iv) having experienced moderate pain intensity (rated as 4 or higher on a 0–10 scale) in the last month. Definite CP was defined as having obvious morphologic features of CP (i.e., Cambridge 3–4 stage or the presence of pancreatic calcifications on computerized tomography scan and/or magnetic resonance cholangiopancreatography, or histologic evidence of CP). Suspected CP group consisted of patients with abdominal pain of 3 or more months duration, 1 episode of acute pancreatitis in the preceding 18 months or recurrent acute pancreatitis who had Cambridge stage 1 or 2 on computerized tomography scan, and/or magnetic resonance imaging/magnetic resonance cholangiopancreatography. Potential participants were excluded if the participant (i) was undergoing treatment for cancer, (ii) was unable to read English well enough to complete questionnaires or the study website, (iii) had current suicidal ideation, or (iv) was currently receiving treatment from a psychologist.

### Trial design and procedures

This pilot feasibility study used a balanced (1:1) randomized parallel-group design. All assessments were completed online through REDCap (17), a secure online data capture platform, and included standardized measures and a 7-day online daily diary to evaluate pain interference and pain intensity. Participants completed assessments pretreatment before randomization, after 8 weeks (immediate posttreatment), and at the 3-month follow-up. There was no examiner bias in outcome assessments because all assessments were completed independently online by the participant.

Randomization was implemented using a computer-generated randomization schedule using blocks of 4 to derive assignments to the 2 treatment conditions (Internet CBT vs control). The randomization schedule was stored in a password-protected document that was only accessible to the study coordinator. The document was formatted to conceal group assignment until the time of randomization after the completion of the pretreatment assessment. Participants were told that they would be offered an Internet pain management intervention either immediately or after 6 months. Participants randomized to the Internet CBT group were provided with immediate access to the *Pancreatitis Pain Course*. Participants randomized to the wait list control group were not provided with access to the program until after the final follow-up assessment was completed (approximately 6 months). Both groups continued to receive usual medical care for CP (which may have included clinic visits and multimodal treatments), which was not altered for this study. Participants received modest gift card incentives for completion of study assessments.

#### Internet CBT condition.

In addition to usual care provided by their treating physician, participants in the Internet CBT group received immediate access to the adapted version of the *Pain Course*, named the *Pancreatitis Pain Course*. The original *Pain Course* is an established Internet CBT program for adults with chronic pain developed by the eCentreClinic and evaluated in several clinical trials to date (15). The program follows a cognitive-behavioral framework where participants learn a range of cognitive and behavioral skills to manage their symptoms and difficulties. Minor content modifications were undertaken to ensure relevance to individuals with CP pain. These included adding brief educational material about CP and adapting case vignettes and examples to represent the symptoms and experiences of patients with CP.

The program includes 5 core online lessons and 5 downloadable lesson summaries, which provide homework assignments to assist participants in learning and applying the skills described in the lessons. These materials are released over 8 weeks (content is metered to allow time for skills acquisition) and include a combination of didactic instruction and narrative examples. Several detailed case stories and real-world examples of individuals with CP pain are integrated throughout the course. The core skills taught in the lessons are shown in Table 1.

**Table 1.** Timetable and content of the pancreatitis pain course

Lesson	Time before next lesson	Lesson content	Primary skill taught
1	1 wk	Education about the prevalence of CP pain and symptoms of anxiety and depression. Information about pain perception and the nervous system. Introduction of a CBT model.	Symptom identification Symptom formulation
2	2 wk	Introduction to basic principles of cognitive therapy and the importance of managing thoughts to help manage pain but also anxiety and depression.	Thought monitoring Thought challenging
3	1 wk	Introduction to physical symptoms of anxiety (i.e. hyperarousal) and depression (i.e., hypoarousal) and their relationship to emotional well-being and pain.	Controlled relaxation Pleasant activity scheduling
4	2 wk	Introduction to behavioral symptoms of anxiety, low mood, and chronic pain. Explanation of the overdoing–underdoing cycle of physical activity and fear and the avoidance of physical activities.	Activity pacing Graded exposure
5	2 wk	Information about the occurrence of lapses in pain, depression, and anxiety. Information about the signs of relapse and the importance of goal setting into the future.	Relapse prevention Goal setting

CBT, cognitive-behavioral therapy; CP, chronic pancreatitis.

Centralized support is available to participants through a weekly coaching call (10–15 minutes by phone). Study coaches were PhD-level postdoctoral psychology fellows with previous experience in CBT for pain management. Coaches encourage participants to practice the skills taught within the course and to gradually adopt them into their everyday lives. To standardize interactions with participants, coaches followed a study coach manual from the *Pain Course* and were supervised by the first author (a licensed clinical psychologist).

Lessons take approximately 20–30 minutes to complete for a total treatment time of approximately 100–150 minutes (treatment content) and 80–120 minutes of coach contact time.

#### Wait list control condition.

Participants assigned to the wait list control condition continued the usual care from their treating physician. After completing the final follow-up assessment, participants in the wait list group were offered the opportunity to receive access to the *Pancreatitis Pain Course*. Fifty percent of participants chose to access the Internet treatment after the follow-up assessment.

### Measures

#### Pretreatment demographic and clinical characteristics.

Participants reported on their age, biological sex, race, employment status, and household annual income. Treating physicians provided information on participants' diagnosis of suspected or definite CP. Participants were identified as using alcohol if they responded yes to the following single item from the Patient-Reported Outcome Measurement Information System v1.0 Alcohol Use—Short Form (18): “In the past 30 days, did you drink any type of alcoholic beverage?” The Patient-Reported Outcome Measurement Information System-29 Profile v2.1 was used to assess depression, anxiety, and sleep disturbance in the past 7 days. Scores > T score of 60 indicate moderate to severe symptoms of depression, anxiety, and sleep disturbance. Good internal reliability and convergent validity have been found in chronic pain samples (19). Finally, participants reported on current opioid medications (e.g., oxycodone) for pain management.

#### Primary outcomes: treatment feasibility and acceptability.

Treatment feasibility was assessed using 3 metrics: (i) study recruitment/enrollment statistics, (ii) treatment engagement as demonstrated by the proportion of participants completing the program, and (iii) number of completed coaching calls.

Treatment acceptability was assessed using qualitative and quantitative metrics. After completing the posttreatment assessment, 9 participants in the Internet CBT group were sequentially invited to participate in a qualitative interview conducted via telephone to evaluate treatment acceptability and satisfaction. This interview was added after the study began and therefore we did not have an opportunity to interview the other 5 participants in this treatment condition. Interviews were recorded and transcribed. For the purpose of this report, we coded responses to 2 questions: (i) “How did the *Pancreatitis Pain Course* help you in managing your pancreatitis pain?” and (ii) “What modifications would you suggest to improve the *Pancreatitis Pain Course*?” These participants also completed the Treatment Evaluation Inventory—Short Form (20) to provide a quantitative rating of treatment acceptability. The Treatment Evaluation Inventory—Short Form includes 9 items and was adapted for this study to be specific to CP pain (e.g., “I find this treatment to be an acceptable way of dealing with chronic pancreatitis pain”). Items are scored on a 5-point scale (1 = strongly disagree, 5 = strongly agree) and summed to create a total score (range 9–45). Scores greater than 27 indicate at least moderate treatment acceptability.

#### Secondary outcomes: pain interference, pain intensity, and quality of life.

Pain interference and pain intensity were assessed using the 24-hour version of the Brief Pain Inventory—Short Form (BPI) (21,22) over 7 days. The pain interference subscale includes 7 items evaluating the impact of pain on sleep, mood, walking ability, general activity, work, relationships, and enjoyment of life over the past 24 hours rated on an 11-point scale from 0 = does not interfere to 10 = completely interferes. Items were summed to create a pain interference total score, and the average total score across all 7 days was used in analyses. The 4 pain intensity items (worst, least, average, and current pain) were rated once daily for 7 days on an 11-point numerical rating scale (0 = no pain, 10 = worst possible pain), and the average pain intensity score across the 7 days was used in analyses. The BPI has been widely used in a variety of populations and has demonstrated strong reliability for detecting changes in pain over time (21,22).

Disease-specific HRQoL was assessed using the Pancreatitis Quality of Life Instrument (PANQOLI) (23), which includes 18 items that map onto 4 subscales (physical function, role function, emotional function, and self-worth) and a total HRQoL score. The PANQOLI has demonstrated good reliability and strong convergent and construct validity among adults with CP (23).

#### Adverse events.

Participants provided open-ended responses concerning adverse events and any significant life events occurring during the trial at the posttreatment and follow-up assessments.

### Sample size

The planned sample size for the trial was 30 subjects based on recommendations for pilot randomized trials that would allow us to test feasibility metrics and preliminary efficacy where 15 subjects per treatment arm allows for the detection of medium (0.5) or large (0.8) effects with 90% power and 2-sided 5% significance (24).

### Data analysis plan

Data analyses were conducted using SPSS v27 (IBM, Armonk, NY). Overall missingness was low, with only 3 individuals with any missing data at posttreatment and 2 at the 3-month follow-up, indicating high feasibility of outcome assessment plan. We used descriptive statistics to summarize the demographic characteristics of the sample.

For our primary aim, we examined the metrics of treatment feasibility and participants' quantitative ratings of treatment acceptability in the Internet CBT group using descriptive statistics. Qualitative interviews were summarized using semantic thematic analysis following the guidelines of Braun and Clarke (25). Two primary coders first reviewed the interview transcripts and then coded the data into meaningful units of text. Next, the 2 coders worked together to group similar codes into categories and then the categories were grouped into overarching themes. At each stage of coding, the codes, categories, and themes were recorded in a code book that included operational definitions and representative quotes. Using this codebook, the 2 coders worked together to achieve consensus. When there was disagreement, a third study team member arbitrated. Using this process, 100% agreement was achieved at each stage of coding.

Our second objective was to explore preliminary treatment effects on pain interference, pain intensity, and disease-specific quality of life. We computed separate ANCOVA models for each outcome measure with group (Internet CBT vs control) as a fixed between-subject factor, and age, sex, and pretreatment scores as covariates. Effect sizes are reported as Cohen *d*, which are interpreted as follows: small effect size = 0.2, medium effect size = 0.5, and large effect size = 0.8 (26). In exploratory analyses, we used the Fisher exact *t* test to compare the proportion of participants in each treatment group who were treatment responders vs nonresponders. As recommended by Patel et al. (27), we created composite responder outcome scores that integrated change scores on pain interference and pain intensity from pretreatment to the 3-month follow-up. Specifically, we classified the proportion of participants achieving at least a 30% (considered moderate improvement) and 50% (considered substantial improvement) (28) or greater reduction in pain interference or pain intensity from pretreatment to the 3-month follow-up. For this pilot feasibility study, we did not impute values for participants with missing data at posttreatment or the follow-up. *P* values <0.05 were considered significant.

## RESULTS

### Participant characteristics

Demographic and clinical characteristics of the sample are summarized in Table 2. Participants were 30 adults with CP (median [M] age = 49.8 years, SD = 12.5; 80% women). Twelve participants had suspected CP and 18 had definite CP. Most participants were not working or employed part-time and represented a range of income levels. At pretreatment, 60% of our sample were taking opioids, 20% had alcohol use, and 47% had moderate to severe symptoms of anxiety, depression, and/or sleep disturbance. Participants recruited from the community were more likely to belong to a racial minority group than participants recruited from clinics (χ^2^ (1) = 5.00, *P* = 0.03); community- and clinic-recruited participants were equivalent on all other demographic characteristics (*P*'s > 0.05).

**Table 2.** Participant demographic and clinical characteristics

	Total sample (n = 30)	Waitlist control (n = 16)	CBT (n = 14)
Age, yr			
Mean (SD)	49.8 (12.5)	49.7 (11.4)	51.6 (14.3)
Range	23–72	26–64	23–72
Gender, n (% women)	24 (80.0)	12 (75.0)	12 (85.7)
Race			
Black (e.g., African, Haitian, Jamaican, Somali)	2 (6.7)	2 (12.5)	0 (0.00)
Chinese and Korean	1 (3.3)	0 (0.0)	1 (7.1)
South Asian (e.g., Indian, Pakistani)	1 (3.3)	0 (0.0)	1 (7.1)
White (Caucasian)	26 (86.7)	14 (87.5)	12 (85.7)
Ethnicity, n (% Hispanic or Latino)	0 (0.0)	0 (0.0)	0 (0.0)
Marital status, n (%)			
Married	21 (70.0)	10 (62.5)	11 (78.6)
Divorced	5 (16.7)	4 (25.0)	1 (7.1)
Single	4 (13.3)	2 (12.5)	1 (7.1)
Employment status, n (%)			
Full-time	10 (33.3)	3 (18.8)	7 (50.0)
Part-time	6 (20.0)	5 (31.3)	1 (7.1)
Not working	14 (46.7)	8 (50.0)	6 (42.9)
Highest level of education completed, n (%)			
High school or less	3 (10.0)	3 (18.8)	0 (0.0)
Vocational or trade school	8 (26.7)	2 (12.5)	6 (42.9)
College or university	13 (43.3)	6 (37.5)	7 (50.0)
Graduate degree/professional school	6 (20.0)	5 (31.3)	1 (7.1)
Annual income, n (%)			
Less than $24,999	5 (16.7)	3 (18.8)	2 (14.3)
$25,000–$49,999	4 (13.3)	4 (25.0)	0 (0.0)
$50,000–$74,999	5 (16.7)	2 (12.5)	3 (21.4)
$75,000–$99,999	4 (13.3)	1 (6.3)	3 (21.4)
$100,000 and above	12 (40.0)	6 (37.5)	6 (42.9)
Pancreatitis diagnosis, n (%)			
Suspected CP	12 (40.0)	6 (37.5)	6 (42.9)
Definite CP	18 (60.0)	10 (62.5)	8 (57.1)
Psychiatric symptoms, n (% *t* score ≥60)			
Depression	12 (40.0)	7 (43.8)	5 (35.7)
Anxiety	11 (36.7)	5 (31.3)	6 (42.9)
Sleep disturbance	14 (46.7)	9 (56.3)	5 (35.7)
Currently using alcohol, n (% yes)	6 (20.0)	3 (18.8)	3 (21.4)
Currently using opioid medication, n (% yes)	22 (73.3)	13 (81.3)	9 (64.3)

CBT, cognitive-behavioral therapy; CP, chronic pancreatitis; SD, standard deviation.

### Feasibility

#### Recruitment/enrollment rate.

**Figure 1.**
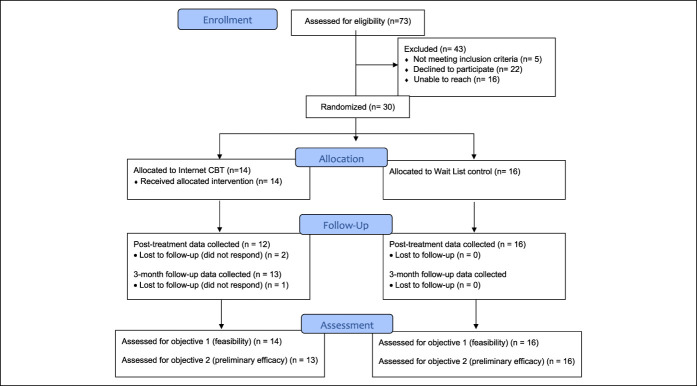
CONSORT flowchart.

Potential participants were recruited sequentially in the order that they were referred and resulted in 73 referrals (33 from clinics and 40 from the community). Sixteen of the referred patients were unable to be reached. Of the 57 potential participants who were reached, 5 did not meet the inclusion criteria (i.e., undergoing treatment for cancer and did not meet CPDPC criteria for suspected or definite CP), and an additional 22 declined to enroll because of lack of time, lack of interest in a psychological treatment, or health complications. The remaining 30 participants (clinic n = 18, community n = 12) enrolled in the study and were included in analyses (overall recruitment/enrollment rate = 53%). Figure 1 shows a CONSORT diagram depicting the flow of study participants through each phase of this pilot feasibility trial.

#### Treatment engagement and adherence.

Initial engagement with the Internet CBT program was high, with 100% of participants completing at least 1 lesson. Engagement with the intervention was good with 64% (n = 9/14) completing all 5 lessons (M = 4.1, SD = 1.4). During treatment, 57% of participants (n = 8/14) completed at least 6 of 8 telephone coaching calls (M = 5.3, SD = 2.3, range = 1–8), which were an average of 10 minutes in duration (range = 5–15 minutes).

### Acceptability

Eighty percent of participants in the CBT group rated the intervention at 27 or higher on the Treatment Evaluation Inventory (M = 29.7, SD = 2.5), indicating moderate to high acceptability of the treatment.

As shown in Table 3, 3 themes emerged in response to how the program was helpful including helpful program features (e.g., program was easy to use and telephone calls from coaches increased accountability), relevance to their own experience with CP pain (e.g., validated own experiences), and helpfulness of skills for coping with pain (e.g., relaxation methods, thought challenging, and activity pacing). Two themes emerged in response to potential modifications to improve the program including increased flexibility of program features (e.g., better visual display on smartphone devices, providing more time to complete lessons) and reflecting a broader range of CP symptoms and life circumstances in case stories.

**Table 3.** Qualitative interviews: main themes

Theme	Example quotes
“How did the *Pancreatitis Pain Course* help you in managing your pancreatitis pain?”	
Helpful program features	“…it did not require any type of computer knowledge to even do, I thought everything about that was very easy.” (participant D02) “I did really like the, you know, review at the- the beginning of each lesson to kind of remember what you'd um learned about uh in the prior lessons.” (participant E11) “I think it's important to keep the follow-up phone calls because of the accountability. I like that accountability, kinda keeps you involved and keeps you reading.” (participant A01).
Relevance to personal experiences living with CP pain	“It is comforting to know that there's other people out there who suffer with this… [the case stories are] just getting the feeling that um, you know, there's other people out there” (participant A08) “The stories kind of included parts of their lives… and how it affected their lives and their family, and then a lot of it again was hearing their mindsets and what works for them and maybe there were little changes of things that they were doing a differently and then oh, maybe I'll try that… or you could turn the information that they were giving you to work for… you, kind of find ways to apply it to your life and kind of translate some of those experiences” (participant A06)
Helpfulness of skills for coping with pain	“It gave me different ways to handle it… I always kind of knew that I was supposed to, you know, do deep breathing and stuff but the course explained more in-depth on how and why that is more important.” (participant A06) “When things got tough then I would have to kind of stop and remind myself okay, now all the sudden you know, I need to challenge these negative thoughts, I need to refocus um you know, my expectations.” (participant D12) “I have done more fun things…spent more time with family and friends. Realizing through the study that it doesn't really make any difference if I'm resting and alone. As far as the pain levels, I may as well be having fun and being social.” (participant A01)
“What modifications would you suggest to improve the *Pancreatitis Pain Course*?”	
Increase flexibility of program features	“I couldn't just go into it from my phone… I kinda had to sit with my computer.” (participant A01) “Space out the time that you had to do your homework a little bit more” (participant A08)
Reflect broader range of symptoms and life circumstances	“[Chronic pancreatitis] is so much more involved than pain, um there's nausea, vomiting, diarrhea, and having to watch everything you eat. ...it would seem a little bit more relatable if they even just mentioned some of the other restrictions around eating and some of the other symptoms.” (participant D02) “It seemed like those people have … either had a lot of free time or they had a lot of flexible work situations… some of it, it is not really geared towards the uh working adult I guess.” (participant G702)

CP, chronic pancreatitis.

### Preliminary efficacy

Means and SDs for our outcome measures by group are presented in Table 4, along with the results of the ANCOVA models for each outcome measure.

**Table 4.** Means and SDs for outcomes by group: ANCOVA models

	Internet CBT: CBT group (n = 14), M (SD)			Wait list control: control group (n = 16), M (SD)			Between groups difference			
	Pretreatment	Posttreatment	3-mo follow-up	Pretreatment	Posttreatment	3-mo follow-up	Posttreatment		3-mo follow-up	
							*P*	*d*	*P*	*d*
Pain interference	3.8 (3.0)	3.6 (2.7)	3.1 (2.3)	4.0 (1.9)	4.5 (2.4)	4.5 (2.5)	0.71	0.15	0.04	0.88
Pain intensity	4.1 (2.3)	3.3 (2.1)	3.4 (2.3)	4.1 (1.8)	4.3 (2.0)	4.2 (1.9)	0.09	0.72	0.07	0.76
Disease-specific quality of life (PANQOLI)										
Total score	53.5 (11.5)	59.6 (13.6)	58.1 (16.8)	58.8 (12.8)	54.8 (11.2)	57.9 (14.1)	0.08	0.72	0.34	0.38
Physical functioning	15.9 (5.4)	16.7 (4.0)	17.2 (7.4)	18.0 (4.8)	17.4 (5.0)	16.2 (6.1)	0.82	0.09	0.23	0.49
Role functioning	15.0 (4.5)	14.3 (4.1)	14.3 (4.3)	13.6 (4.0)	12.9 (4.4)	15.3 (4.3)	0.46	0.30	0.39	0.35
Emotional functioning	9.6 (3.1)	12.4 (5.1)	11.2 (5.8)	13.1 (3.9)	10.6 (4.2)	12.2 (4.0)	0.18	0.55	0.37	0.36
Self-worth	13.0 (4.1)	16.0 (5.1)	15.3 (5.3)	14.1 (5.0)	13.8 (3.8)	14.3 (5.0)	0.11	0.66	0.32	0.40

CBT, cognitive-behavioral therapy; CP, chronic pancreatiitis; M, median; PANQOLI, Pancreatitis Quality of Life Instrument.

#### Pain interference.

From pretreatment to immediate posttreatment, change in pain interference on the prospective 7-day BPI was similar for participants in the Internet CBT and control groups, *F* (1, 27) = 0.14, *P* = 0.71, and *d* = 0.15. However, at the follow-up, the Internet CBT group reported significantly greater reductions on pain interference compared with the control group, and this was a large effect size, *F* (1, 28) = 4.92, *P* = 0.04, and *d* = 0.88.

#### Pain intensity.

Participants in the Internet CBT group reported greater reductions in pain intensity than the control group through posttreatment and follow-up. These were moderate to large effect sizes (*F* (1, 27) = 3.15, *P* = 0.09, *d* = 0.72; *F* (1, 28) = 3.65, *P* = 0.07, *d* = 0.76, respectively).

#### Disease-specific HRQoL.

The Internet CBT group had a greater improvement in PANQOLI Total score compared with the control group from pretreatment to posttreatment, and this was a moderate effect size, *F* (1, 28) = 3.31, *P* = 0.08, *d* = 0.72. Treatment group differences in PANQOLI Total scores lessened at the follow-up (*F* (1, 29) = 0.94, *P* = 0.34, *d* = 0.38). Between-group differences on the PANQOLI Emotional Functioning, Self-Worth, Physical Functioning, and Role Functioning subscales were not statistically significant at immediate posttreatment or follow-up, and these were small effects.

#### Treatment responders vs nonresponders.

**Figure 2.**
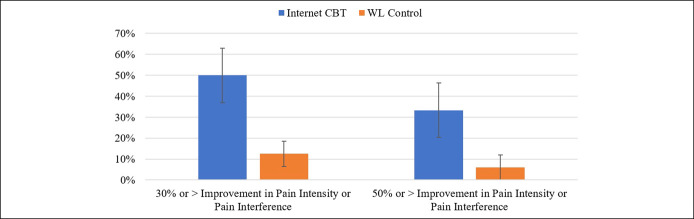
Treatment responders by group. CBT, cognitive-behavioral therapy; WL, waitlist.

As shown in Figure 2, χ^2^ analysis indicated that the proportion of participants who achieved at least a 30% reduction in pain interference or pain intensity from pretreatment to the 3-month follow-up was significantly greater in the Internet CBT group than the control group (50% vs 13%, Fisher exact *t* test *P* = 0.04). The Internet-CBT group also had a higher proportion of participants who achieved a 50% reduction in pain or pain interference from pretreatment to the 3-month follow-up (33.3% vs 6.3%, Fisher exact *t* test *P* = 0.09).

#### Adverse events.

No serious adverse events occurred during the study. Major family and health events (e.g., hospitalization, surgery, stroke, and death of family member) were reported by 7 participants (n = 4 in the CBT group, n = 3 control group) during the study period. However, these were not described or categorized as study-related adverse events.

## DISCUSSION

Our primary aim was to conduct the first trial of a remotely delivered CBT pain self-management intervention for adults with painful CP and examine its feasibility and acceptability through a pilot RCT. We adapted an established program that had already shown effectiveness in other pain populations (15,29) to be relevant for adults with CP. Findings confirmed feasibility and acceptability using several metrics, including achieving an adequate recruitment and participation rate, achieving moderate to high acceptability ratings from users, and relatively high treatment completion rates. Qualitative interviews provided additional participant perceptions of the treatment, suggesting that most individuals felt that the program was easy to use, relevant, and helpful for coping with painful CP.

Our secondary aim was to explore the effects of Internet CBT on pain, pain interference, and quality of life. We found significant reductions in pain intensity and pain interference in participants receiving the *Pancreatitis Pain Course* compared with those in the wait list control condition, supporting our hypotheses and extending knowledge of CBT effects to this population. Using a well-researched cutoff of 30% reduction as representing moderately important improvements (28) in pain intensity or pain interference from baseline to the 3-month follow-up, we found that 50% of the treatment group achieved improvement compared with only 13% of the wait list control group. The same pattern of findings was also found when a more stringent 50% reduction cutoff was used, with 33% and 6% in the treatment and control groups meeting the criteria, respectively.

The *Pancreatitis Pain Course* equips patients with strategies that directly address cognitive, emotional, social, and behavioral factors to minimize the impact of their painful CP on their activity limitations and overall well-being. Our findings extend the evidence base of CBT, which has been extensively studied in other chronic pain conditions (9). In the short-term, we have preliminary evidence that our pain self-management program is feasible, acceptable, and leads to symptom reduction (reduced pain and pain interference) among persons with CP. Larger RCTs using rigorous designs and adequate sample sizes are needed to confirm this finding and to examine long-term outcomes (e.g., 12 months postintervetnion) on psychological functioning, HRQoL, and healthcare use and costs (e.g., indirect and direct costs of pain and disease-related treatments). It will also be important to study the applicability of the *Pain Course* to other gastrointestinal conditions associated with chronic pain (e.g., irritable bowel syndrome, inflammatory bowel disease).

Given that previous pain management interventions in CP have focused almost exclusively on pharmacological and surgical approaches (e.g., (30,31)), applying CBT for painful CP is very novel. There has been 1 promising pilot study of a phone-based mindfulness service that was also shown to be feasible and potentially useful for painful CP (10). Recent guidelines for managing painful CP (8) have encouraged better aligning approaches to pain management with the multidimensional nature of chronic pain, recognizing the component of central sensitization in CP demonstrated in previous studies. Conclusions from multiple systematic reviews and meta-analyses of CBT interventions for chronic pain (9) are that pain self-management should be considered as part of standard care for all adults with chronic pain. By delivering CBT using an Internet program, we also address patient barriers including having limited access because of difficulties with transportation, direct and indirect costs of therapy visits, caregiver responsibilities, health and mobility, scheduling, and time commitment, which has also been recognized as important considerations for telehealth for gastroenterology practice (32). Moreover, if a program is made publicly accessible, barriers pertaining to practice setting (e.g., academic vs community/private practice) can also be addressed. Remote delivery of pain self-management treatments, as explored in this study, are also increasingly being recognized as a viable way of improving access to these treatments for large numbers of patients (33).

Our findings should be interpreted in light of several limitations. Because we did not include a measure of treatment expectancy or an attention control comparator, we are unable to know the influence of expectancies or to separate treatment from placebo effects. However, given that this is the first trial of CBT for painful CP, we chose a design that prioritized understanding feasibility and acceptability of the intervention. Future studies with a placebo attention condition are needed to definitely evaluate treatment effects. Our sample is small, predominantly women, mostly college educated, and mostly White. Although the addition of community recruitment helped to increase racial diversity, our findings may not generalize to samples with greater socioeconomic and racial diversity. Future studies are needed with larger samples and using a broader range of outcomes over a longer duration to examine the durability of treatment effects (34). Given that most of our sample was using opioids at baseline for pain management and benefits are reported for the *Pain Course* in other pain populations in decreasing opioid use (35), this will be an important outcome to evaluate in future trials.

In conclusion, we present the results from the first trial of a pain self-management program adapted for adults with CP pain. Future research to extend these findings in a larger and more definitive RCT using an attention-control group are needed. Ultimately, if successful, remotely delivered interventions, such as the *Pancreatitis Pain Course*, could be widely disseminated to effectively support pain management among persons with CP.

## CONFLICTS OF INTEREST

**Guarantor of the article:** Tonya M. Palermo, PhD.

**Specific author contributions:** T.M.P. and M.D.T.: conceived and designed the study. M.D.T., S.S.V., E.F., G.T., D.L.C., and D.Y.: reviewed participants for study eligibility. T.M.P. and B.F.D.: adapted the intervention. K.S. and Y.J.K.: performed the qualitative interviews and analysis. E.F.L.: conducted the data analysis. T.M.P. and E.F.L.: drafted the manuscript. B.F.D., D.L.C., D.Y., and D.K.A.: provided critical revisions for important intellectual content. All authors revised the manuscript and approved the final version.

**Financial support:** Research reported in this publication was supported by the National Cancer Institute and National Institute of Diabetes and Digestive and Kidney Diseases of the National Institutes of Health under award numbers U01 DK108306 (D.Y.), U01 DK108323 (E.F.), U01 DK126300 (G.T.), U01 DK108327 (D.L.C.), and U01 DK108288 (M.D.T., S.S.V., and T.M.P.). E.F.L. was supported by K23NS089966 from the National Institute of Neurologic Disorders and Stroke of the National Institutes of Health. K.S. was supported by a University of Washington Mary Gates Research Scholarship Award. B.F.D. was supported by an Australian NHMRC Career Development Fellowship. The content is solely the responsibility of the authors and does not necessarily represent the official views of the National Institutes of Health.

**Potential competing interests:** T.M.P., E.F.L., M.D.T., K.S., Y.J.K., S.S.V., E.F., G.T., D.K.A., D.L.C., and D.Y. have no conflicts of interest to declare. B.F.D. is a developer of the Pain Course, but derives no personal or financial benefit from it.

**Clinical trial registration:** ClinicalTrials.gov; Identifier: NCT03322644.

**Study Highlights**

**WHAT IS KNOWN**

✓ Severe abdominal pain is common in patients with chronic pancreatitis (CP).
✓ Few nonpharmacological pain interventions have been evaluated for painful CP.
✓ Cognitive-behavioral therapy (CBT) has been shown efficacious for reducing pain and disability for other chronic pain conditions.

**WHAT IS NEW HERE**

✓ Conducted the first trial of CBT pain self-management for painful CP.
✓ CBT delivered through the Internet was feasible and acceptable to adults with suspected and definite CP.
✓ Participants in the CBT condition had greater reductions in pain intensity and pain interference than those in the control condition.

**TRANSLATIONAL IMPACT**

✓ Internet-delivered cognitive-behavioral therapy shows promise for reducing pain and disability in patients with painful CP and has potential for wide dissemination.
✓ Technology-delivered interventions could address barriers to receiving psychological services in person in gastroenterology practices.
